# Stroke Lesion Outcome Prediction Based on MRI Imaging Combined With Clinical Information

**DOI:** 10.3389/fneur.2018.01060

**Published:** 2018-12-05

**Authors:** Adriano Pinto, Richard Mckinley, Victor Alves, Roland Wiest, Carlos A. Silva, Mauricio Reyes

**Affiliations:** ^1^CMEMS-UMinho Research Unit, University of Minho, Guimarães, Portugal; ^2^Centro Algoritmi, University of Minho, Braga, Portugal; ^3^Support Center for Advanced Neuroimaging, University Institute for Diagnostic and Interventional Neuroradiology, Inselspital, Bern University Hospital, Bern, Switzerland; ^4^Institute for Surgical Technology and Biomechanics, University of Bern, Bern, Switzerland

**Keywords:** stroke, machine learning, deep learning, MRI, prediction

## Abstract

In developed countries, the second leading cause of death is stroke, which has the ischemic stroke as the most common type. The preferred diagnosis procedure involves the acquisition of multi-modal Magnetic Resonance Imaging. Besides detecting and locating the stroke lesion, Magnetic Resonance Imaging captures blood flow dynamics that guides the physician in evaluating the risks and benefits of the reperfusion procedure. However, the decision process is an intricate task due to the variability of lesion size, shape, and location, as well as the complexity of the underlying cerebral hemodynamic process. Therefore, an automatic method that predicts the stroke lesion outcome, at a 3-month follow-up, would provide an important support to the physicians' decision process. In this work, we propose an automatic deep learning-based method for stroke lesion outcome prediction. Our main contribution resides in the combination of multi-modal Magnetic Resonance Imaging maps with non-imaging clinical meta-data: the thrombolysis in cerebral infarction scale, which categorizes the success of recanalization, achieved through mechanical thrombectomy. In our proposal, this clinical information is considered at two levels. First, at a population level by embedding the clinical information in a custom loss function used during training of our deep learning architecture. Second, at a patient-level through an extra input channel of the neural network used at testing time for a given patient case. By merging imaging with non-imaging clinical information, we aim to obtain a model aware of the principal and collateral blood flow dynamics for cases where there is no perfusion beyond the point of occlusion and for cases where the perfusion is complete after the occlusion point.

## 1. Introduction

Stroke ranks second as leading cause of death worldwide (1), with ischemic stroke being the most common type (2). Ischemic stroke arises from an artery occlusion caused by local thrombolysis, hemodynamic factors or embolic causes. Due to artery occlusion, the surrounding area suddenly suffers a blood flow reduction, leading the cells to a transient state slightly above cell death. The hypo-perfused area concerns the tissue at risk, also known as salvageable tissue, that can eventually reach a non-viable point of failure even after flow restoration (3, 4). Therefore, stroke lesion can be characterized by a core tissue, encompassed by brain dead tissue, and a penumbra tissue corresponding to the salvageable tissue. The temporal evolution of a stroke lesion can be characterized into four main phases: hyper-acute (initial event), acute (6 h after event), sub-acute (from 24 h) and chronic phase (from 2 weeks) (5).

Neuroimaging plays an essential role in the diagnosis and treatment of stroke, where Computed Tomography (CT) and Magnetic Resonance Imaging (MRI) are the preferred imaging modalities. However, MRI provides a better detection and assessment of potentially salvageable tissue, due to its multi-spectral property (6). After diagnosing and evaluating the stroke lesion through neuroimaging acquisitions, the clinicians need to plan the treatment phase. Such phase encompasses either mechanical thrombectomy or thrombolysis (7, 8) to revascularize the hypo-perfused tissue, which is only viable for the sub-acute phase. Therefore, in a short period of time, expert physicians must carefully evaluate the associated risks and benefits of the clinical intervention, namely the volume of hypo-perfused tissue potentially salvageable vs. the risk of causing haemorrhage or other complications (7, 9). If performed, the reperfusion success is assessed via the standardized Thrombolysis in Cerebral Infarction (TICI) scale (9).

Predicting stroke lesion outcome (i.e., at 3-month follow-up), and the potential efficacy of the treatment according to the nature of the lesion, has a great potential to guide the decision making of physicians. An automatic stroke tissue outcome prediction method would help the physician in such time-critical decision-making process (10). In this paper, we propose a novel end-to-end deep learning architecture that combines imaging information with clinical meta-data, the TICI scale. Our method incorporates clinical meta-data at two levels. First, at the population level, which implicitly encodes expected correlations between tissue loss and the TICI score into a custom loss function of the network. Second, at a patient level, which explicitly encodes the TICI score of each patient as an extra input channel of the network. To evaluate our proposal, we used the publicly available ISLES 2017 dataset, where we show the potential value of incorporating imaging and clinical meta-data for stroke tissue outcome prediction at a 3-month follow-up.

### 1.1. Previous Work

Several methods have been proposed for stroke lesion segmentation (11). However, only recently approaches based on machine learning have been proposed for ischemic stroke lesion outcome prediction. These proposals are based on multivariate linear regression models (12–14), decision trees (15), and CNN-based deep learning architectures (16, 17).

Scalzo et al. (12) proposed a framework to predict stroke tissue outcome, 4 days after clinical intervention (thrombectomy), based on Fluid Attenuation Inversion Recovery (FLAIR) MRI sequence, and Apparent Diffusion Coefficient (ADC) and Time-to-Maximum (Tmax) maps, if available. Tissue outcome prediction was achieved through a regression model that learns the behavior of neighbouring voxels within a cuboid. Kemmling et al. (14) used CT and MRI perfusion maps alongside clinical information, encompassing the reperfusion success. The authors used a generalized linear model to consider the effect of multiple clinical variables when performing the stroke lesion outcome prediction, however, each voxel is considered independently, disregarding spatial context. Rose et al. (13) proposed a two-stage approach for stroke lesion outcome prediction based on perfusion maps, Cerebral Blood Flow (CBF), Cerebral Blood Volume (CBV), Mean Transit Time (MTT), and Diffusion-Weighted Imaging (DWI) maps. Initially, the method defines a region of interest (ROI) from the intensity signal of the perfusion and diffusion maps. Afterwards, a Gaussian mixture model, trained in different sets of MRI maps, performs stroke outcome prediction. McKinley et al. (15) also used a two-stage classification, where each stage comprehends two Random Forests (RFs). In the first stage, the method focusses on lesion delineation, through the definition of a ROI, where each classifier considers features extracted from different sets of MRI maps. After defining the hypo-perfused ROI, a second set of two RFs performs a precise prediction of the stroke lesion. These classifiers are trained on different sets of patients. One classifier is trained with patients with no reperfusion, to obtain worst case scenarios, whereas a second classifier is trained in patients with good reperfusion, therefore predicting scenarios where hypo-perfused tissue has higher chances of being salvaged. Afterwards, the final prediction is obtained by combining the results of both classifiers, using a logistic regression model.

Most recent methods are based on deep learning. Choi et al.(17) employed an ensemble of 12 deep learning methods, divided in two different groups. One group performs voxel-wise segmentation, based on the U-net architecture (18) adapted for 3D data, totalling four models. The other group encompasses Fully Connected Networks architectures with different patch sizes, to perform classification. The final prediction results from a weighted merging.

In previous approaches, the clinical information related to the success of reperfusion (TICI scale) has either been used within multivariate linear regression models (14), or to dichotomize the training data to train specific RFs models (15). Nonetheless, non-imaging clinical information has up to our knowledge not been integrated in deep learning architectures to predict stroke lesion outcome.

### 1.2. Contributions

In this paper, we propose an automatic method for stroke lesion outcome prediction, whose main contributions are:

1. The combination of imaging and non-imaging clinical data in an end-to-end deep learning architecture.
2. The development of a customized loss function to incorporate clinical information during the learning phase. Therefore, learning relationships between imaging and non-imaging information at a population level.
3. The inclusion of clinical information during the prediction phase at a patient-specific level, allowing us to perform predictions of different outcome scenarios in clinical environment.

The following sections are organized as follows: section 2 describes the proposed method. Section 3 details the database used and evaluation methods. Section 4 presents the results and its discussion. Finally, section 5 summarizes up the main aspects of the proposal.

## 2. Methods

Stroke lesion outcome prediction consists of characterizing follow-up changes in location and extension of lesions over time from multi-sequence MRI and clinical information. In our proposal, to perform tissue outcome prediction, the method assigns to each voxel of the MRI volume one out of two classes, healthy tissue or stroke lesion. The following subsections describe the main steps of our proposal.

### 2.1. Pre-Processing

**Figure 1:**
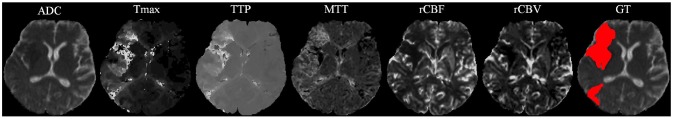
MRI parametric maps of a stroke patient with TICI score 0, and the respective manual segmentation. Only one class is defined, describing simultaneously the infarct core and the penumbra regions.

**Figure 2:**
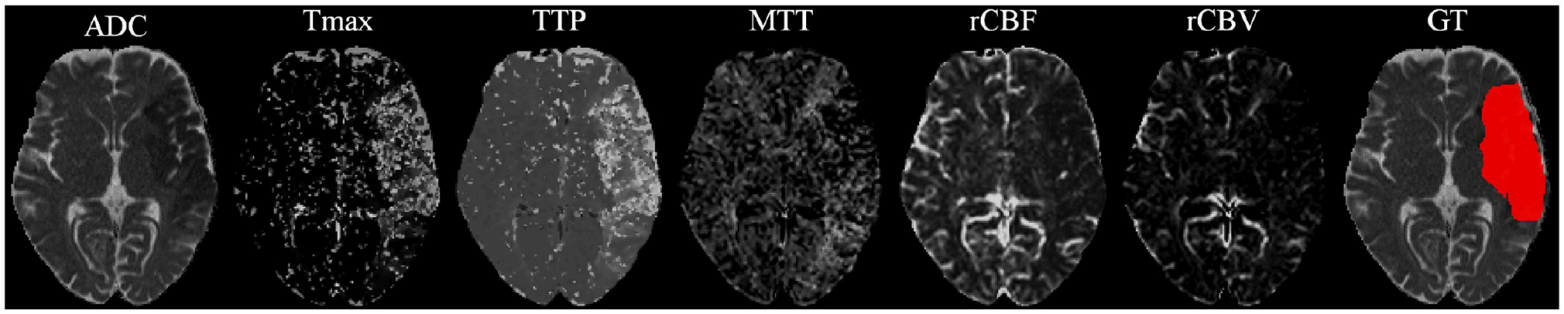
MRI parametric maps of a stroke patient with TICI score 3, and the respective manual segmentation.

Our proposal uses diffusion and perfusion maps, adding up to six MRI parametric maps: diffusion ADC map, and perfusion relative Cerebral Blood Flow (rCBF), relative Cerebral Blood Volume (rCBV), Mean Time to Transit (MTT), Time-to-Peak (TTP), and Tmax maps. Figures 1, 2 show two cases of MRI maps with different TICI scores, alongside the manual segmentation (ground truth) obtained from a T2 sequence at a 90 day follow-up.

ISLES 2017 dataset provides MRI acquisitions from different centers (19). So, the perfusion and diffusion maps were acquired with different sets of configurations. Therefore, for each patient we first resized all maps to a common volume of dimension of 256 × 256 × 32. Afterwards, the ADC maps were clipped between [0, 2, 600] × 10^−6^*mm*^2^/*s* and the Tmax maps were clipped to [0, 20*s*], since values beyond these ranges are known to be biologically meaningless (15). As a final step of pre-processing, we applied a linear scaling across all maps transforming them to the range [0, 255].

### 2.2. Deep Learning Architecture

Deep learning encompasses a variety of representation learning techniques capable of automatically learning hierarchical and complex features from the data. This property grants various levels of abstraction, translating to higher discriminative features, when comparing to hand-crafted features. In imaging processing, the most common techniques of deep learning are the Convolutional Neural Networks (CNNs) (20–22) and the Recurrent Neural Networks (RNNs) (22, 23).

CNNs have recently achieved remarkable success in well-known computer vision challenges (21). CNNs convolve a set of kernels over an input (image or image patches) obtaining a new feature space that characterizes local interactions in the input data.

Gated RNNs, which achieved success in the biomedical imaging field (23), provide a tighter notion of context. Initially proposed for the analysis of discrete sequences, their architecture contains gates that learn to store and read information from linear units. Due to this property, Gated RNNs, namely Long-Short Term Memory (24) and Gated Recurrent Unit (25), can process inputs and outputs of varying lengths and retain information over long time-steps. When applied to computer vision, the memory capability of multi-dimensional gated RNNs allows us to model interactions among all the input data, which translates to a higher notion of context regardless of the receptive field.

**Figure 3:**
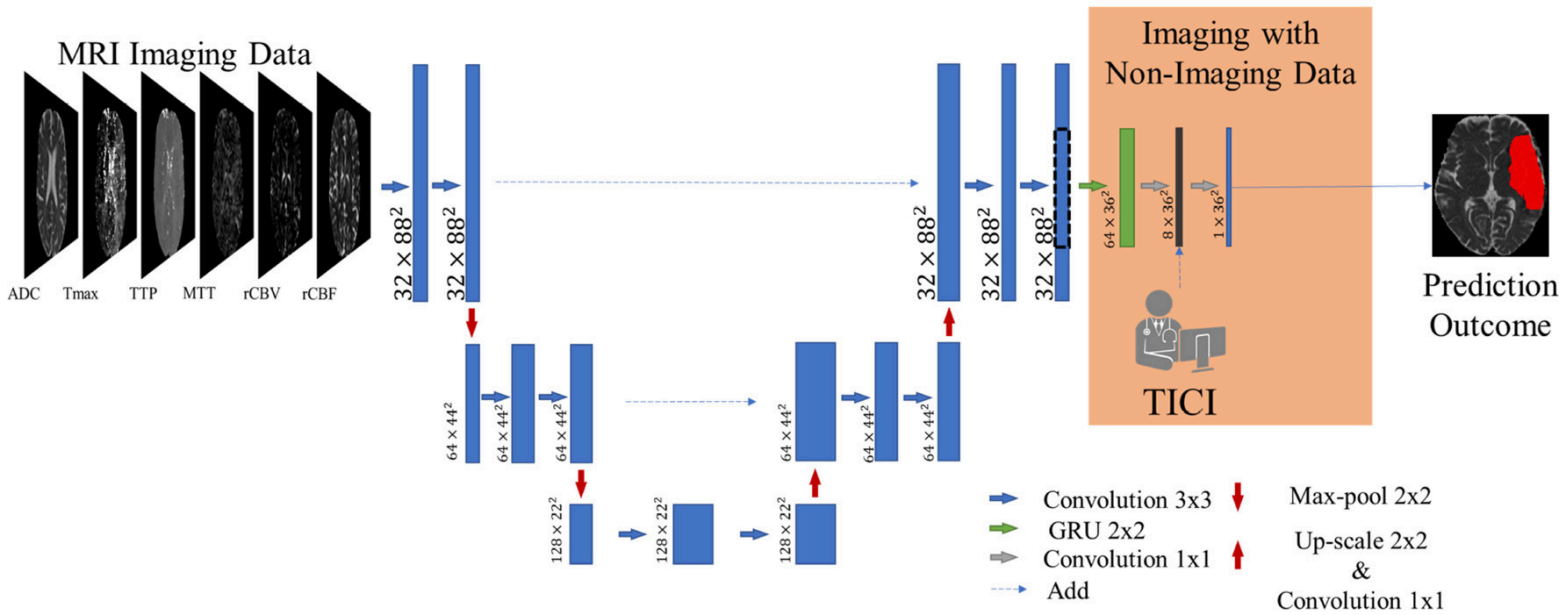
Overview of the proposed architecture. Blue feature maps result from 2D-dimensional convolutions. The green feature maps represent the 2D-dimensional GRU layer. The first dimension corresponds to the number of feature maps. The dashed line consists of a cropping step to connect the U-Net with the GRU layer. The prediction is provided by the last layer, corresponding to a SoftMax activation.

Our proposal is inspired by the fully convolutional U-net architecture (18), which has proved to be competitive in many biomedical image segmentation applications. In addition, we combined the U-net with a 2D-dimensional Gated Recurrent Unit (GRU) layer (25) to obtain smoother and structured predictions. Figure 3 shows the proposed architecture. The convolutional layers are responsible for the generation of discriminative feature vectors. Afterwards, the feature maps are fed into the GRU layer to enforce the spatial context of the network. Finally, a convolutional layer of 1 × 1 reduces the feature space to combine it with the clinical information.

### 2.3. Combining Imaging With Non-imaging Data

Besides MRI imaging data, non-imaging clinical information is also gathered during the acute phase of stroke, such as the Time Since Stroke (TSS), Time to Treatment (TTT), modified Ranking Scale (mRS) score, and TICI score. TSS and TTP are time measures that mark the time-points when the stroke incident was diagnosed and when clinical intervention was performed, respectively. The mRS score characterizes the degree of disability 90 days after a stroke incidence. However, the most relevant factor is the TICI score (9), which indicates the degree of success of the mechanical thrombectomy, based on cerebral angiography. Low scores (TICI ∈{0, 1}) describe cases with minimal perfusion or no perfusion at all. Mid-range scores (TICI ∈{2*a*, 2*b*}) characterize cases with progressively better partial perfusion. The highest score (TICI = 3) characterizes a complete flow-restoration (9). Consequently, it is expected that higher TICI scores naturally lead to increased levels of tissue being salvaged, and conversely, lower TICI scores might indicate increased levels of tissue loss. In our proposal, we aim to incorporate this information into a deep learning architecture, to relate imaging (e.g., stroke location, extension) with clinical information. In our, proposal we aim to include such knowledge during the learning and testing phases of the system. To do so, our method considers the TICI scale at two levels: population-level and patient-level.

#### 2.3.1. Population-Level

Incorporating clinical information at a population-level is achieved through a custom loss function, which drives the learning process to solutions conditioned to the clinical TICI score. Due to the presence or absence of perfusion beyond the location of the occlusion, stroke lesion extension can present changes between the TSS and the follow-up acquisitions. For cases with no perfusion, it is expected that the lesion grows between the two exams, while cases with existent perfusion should present a shrinkage of the lesion volume. In our proposal, we aim to model such lesion dynamics when predicting the lesion progression from the MRI parametric maps at the first exam to a future time. To do so, the training procedure is performed based on the MRI sequences from the first exam and the manual segmentation of the lesion at the follow-up acquisition. When the lesion shrinks, our system must learn that although the lesion presents a larger extension in the MRI sequences, it should produce a smaller segmentation, and when the lesion grows, it should learn to predict a larger segmentation, although the information provided by the MRI sequences indicates it is smaller. We may model this dynamic by interpreting the growth as oversegmentation, and the shrinkage as undersegmentation in relation to the information supported by the MRI sequences in the present time. We may interpret the oversegmentation as an increase in false positives (FP) and the shrinkage as an increase in false negatives (FN), since these are not supported by the information in the MRI sequences, acquired at the first medical exam. Such dynamic in our proposal is modeled by the *F*_β_ score that combines the Precision and Recall scores as follows:

(1) $$F_{\beta }=(1+\beta^{2})\frac{precision\times recall}{(\beta^{2}\times precision)+recall}.$$

The Precision score, defined as $Precision=\frac{TP}{TP+FP}$, measures the presence of false positives (FP), while the Recall, given by $Recall=\frac{TP}{TP+FN}$, considers the presence of false negatives (FN) (TP corresponds to the number of true positives). As shown in Equation (1), the relation between these two scores is controlled by β, which in our proposal encodes the TICI score. To be applicable to a supervised learning approach, *F*_β_ needs to relate the predictions with the ground truth, which is defined in the following way:

(2) $$F_{\beta }=(1+\beta^{2})\frac{\sum_{i}^{N}p_{i}g_{i}}{\sum_{i}^{N}\beta^{2}p_{i}^{2}+\;\sum_{i}^{N}g_{i}^{2}}.$$

The sum is performed for the *N* voxels of the patch in the prediction, *p*_*i*_∈*P*, and the ground truth, *g*_*i*_∈*G*. The gradient of the *F*_β_ score for the *j*^*th*^ voxel prediction is computed as:

(3) $$\frac{\delta F_{\beta }}{\delta p_{j}}=(1+\beta^{2})\left(\frac{g_{j}(\sum_{i}^{N}\beta^{2}p_{i}^{2}+\;\sum_{i}^{N}g_{i}^{2})-(2\beta^{2}p_{j})\sum_{i}^{N}p_{i}g_{i}}{{\left(\sum_{i}^{N}\beta^{2}p_{i}^{2}+\;\sum_{i}^{N}g_{i}^{2}\right)}^{2}}\right).$$

#### 2.3.2. Patient-Level

The inclusion of the TICI score at a patient level is achieved by an extra channel before the final layer of the architecture (see Figure 3). By combining the feature set extracted from imaging data and the respective TICI score, we aim to drive the learning process to search for correlations among them. With this approach we hypothesize that the model should be aware that different TICI scores should predict different lesion outcomes, during the estimation phase. Therefore, our proposal would be capable of predicting the amount of salvageable tissue loss in the presence and absence of recanalized perfusion.

### 2.4. Post-Processing

As post-processing step, we performed simple morphological filtering. Stroke lesions vary significantly in size. The post-processing should take this variation into account to avoid the complete removal of stroke lesions; therefore, a threshold to remove only connected components with less than 25 voxels was defined using cross-validation.

## 3. Experimental Setup

We evaluated our proposal on the ISLES 2017 training and testing datasets, where the online platform also includes an automated evaluation of prediction results submitted to the system. In this work, we compared the performance of our proposal with and without using clinical meta-data.

### 3.1. Dataset

ISLES 2017 dataset comprises a total of 75 ischemic stroke patients divided into two groups: training (*n* = 43) and testing (*n* = 32), who underwent mechanical thrombectomy. For each subject a total of six MRI acquisitions are provided: ADC, TTP, Tmax, rCBV, and rCBF. All image modalities are already co-registered and skull-stripped (16). Alongside the diffusion and perfusion parametric MRI maps, each patient has a lesion outcome manually segmented by a clinician on a 90-day follow-up T2 MRI. The ground truth was provided only for the training dataset, since the test set is evaluated by the online platform. Alongside the imaging information, each patient is also characterized by the TICI score, TSS, TTT, and mRS Score. Although other clinical information is available, only the TICI scores were used in this study. Table 1 describes the distribution of TICI score for each available dataset.

**Table 1:** TICI distribution for ISLES 2017 training and testing datasets.

	**TICI 0**	**TICI 1**	**TICI 2a**	**TICI 2b**	**TICI 3**
Training	6 (14%)	3 (7%)	3 (7%)	11 (26%)	20 (46%)
Testing	3 (9%)	2 (6%)	4 (13%)	6 (19%)	17 (53%)

### 3.2. Evaluation

The performance of each method was evaluated using five metrics: Dice Similarity Score (DSC), Precision, Recall, Hausdorff Distance and Average Symmetric Surface Distance (ASSD). DSC measures the similarity between two volumes and is defined by $DSC=\frac{2TP}{FP+2TP+FN}$. As for the distance metrics, Hausdorff Distance denotes the maximum distance between two volumes surface points, capturing outliers. It is defined as: $HD\left(A,B\right)=\text{max}\left\{\underset{a\in A}{\max}\underset{b\in B}{\min}d\left(a,b\right),\underset{b\in B}{\max}\underset{a\in A}{\min}d\left(b,a\right)\right\}$. Finally, ASSD describes the average distance between the volumes surface points defined as: $ASSD\left(A,B\right)=\frac{\underset{a\in A}{\sum }min_{b\in B}d\left(a,b\right)}{\left|A\right|}$.

### 3.3. Setup

The validation set comprised seven cases, while the testing set of 36 cases from ISLES 2017 training set. To assess the added value of our contributions, we perform a 7-fold-cross-validation scheme within the training set. We compare our proposal with a baseline architecture, which does not encompass any clinical meta-data. In addition, we changed the loss function to the soft dice (26), which is a standard loss function for segmentation tasks.

### 3.4. Hyper-Parameters

For each subject, around 500 patches of size 88 × 88 were extracted, using a uniform random sampling scheme. The network was trained with ADAM optimizer (27) (learning rate of 1 × 10^−5^) using a mini-batch size of 4. The implementation was based on Keras (28) with Theano backend. All tests were conducted on a workstation equipped with a GeForce GTX 1070 with 8 GB. For each patient, prediction took around 15s.

#### 3.4.1. Inclusion of Clinical Information

When considering cases with low TICI score, predicting the maximal extent of tissue loss eases the clinical decision-making process, therefore decreasing the chances of tissue death by hypo-perfusion. In such circumstances, with the inclusion of the TICI score we aim to drive the model to predict the worst-case scenario of stroke lesion outcome. Conversely, in a case with a high TICI score we would prefer a prediction where the recovered hypo-perfused tissue due to reperfusion is achieved with success, holding on the same principles as before. It is worth mentioning that such relationship is further affected by several other clinical and patient-specific pathophysiological aspects, such as collateral flood, onset time of the stroke, etc.

Giving the available number of cases per TICI in ISLES 2017 dataset, we merged TICI scores, increasing the number of cases per score. Therefore, at a population level, β in Equation (4) encodes the TICI score as follows:

(4) $$\beta =\{\begin{array}{ll} 2, & \text{if }TICI\in \left\{0,1\right\}\\ 1, & \text{if }TICI\in \left\{2,2a,2b\right\}\\ 0.5, & \text{if }TICI=3 \end{array}$$

In this way, for *TICI* = 3 (i.e., complete perfusion) we defined β = 0.5, so recall is weighted four times less than precision. Hence, we drive the model to give higher importance to the expression of false positives rather than false negatives, preferring scenarios with low tissue loss. Conversely, for TICI ∈{0, 1} (i.e., poor recanalization), we defined a β = 2, where recall is weighted four times higher than precision. For such cases, the motivation is to give preference to high tissue loss. Finally, for TICI ∈{2*a*, 2*b*} the value of β = 1, obtaining the Dice Score, where precision and recall are equally taken into consideration. Such scale of β was defined through cross-validation.

## 4. Results and Discussion

In this section, we first evaluate the main contribution of our proposal in the training set. Using cross-validation we compare the performance of the baseline method without non-imaging clinical information against our proposal. Afterwards, we present the results obtained in ISLES 2017 testing dataset, performing a comparison against state-of-the-art methods.

### 4.1. Incorporation of Non-imaging Clinical Information

Due to the large diversity of appearance, size and shape, the tissue outcome prediction presents as a challenging task (10). In this study, we show the importance of having non-imaging clinical information in a neural network, to characterize principal and collateral blood flow hemodynamic and obtain better prediction outcomes. The results obtained for the training set are shown in Table 2.

**Table 2:** Results obtained through cross-validation in ISLES 2017 training dataset for the baseline method and our proposal. Each metric contains the average ± standard deviation.

	**Dice**	**Hausdorff distance**	**ASSD**	**Precision**	**Recall**
Baseline	0.34 ± 0.22	35.09 ± 17.27	6.08 ± 5.27	0.37 ± 0.29	0.54 ± 0.26
Proposal	0.35 ± 0.22	31.38 ± 15.81	5.55 ± 5.00	0.41 ± 0.30	0.47 ± 0.24

When comparing with the baseline, our proposal is capable of achieving higher DSC and lower Hausdorff Distance, showing the added value of incorporating the TICI score into the neural network. Considering the precision and recall metrics, our proposal achieved higher precision but lower recall. This suggests a higher capability to perform stroke lesion outcome prediction, by depicting gradual changes in the hypo-perfused tissue. We hypothesize that making the model aware to intrinsic biological phenomena of lesion growth or shrinkage (TICI dependent) lead to more precise predictions, which is sustained by the lower values of distance metrics and higher DSC score.

**Figure 4:**
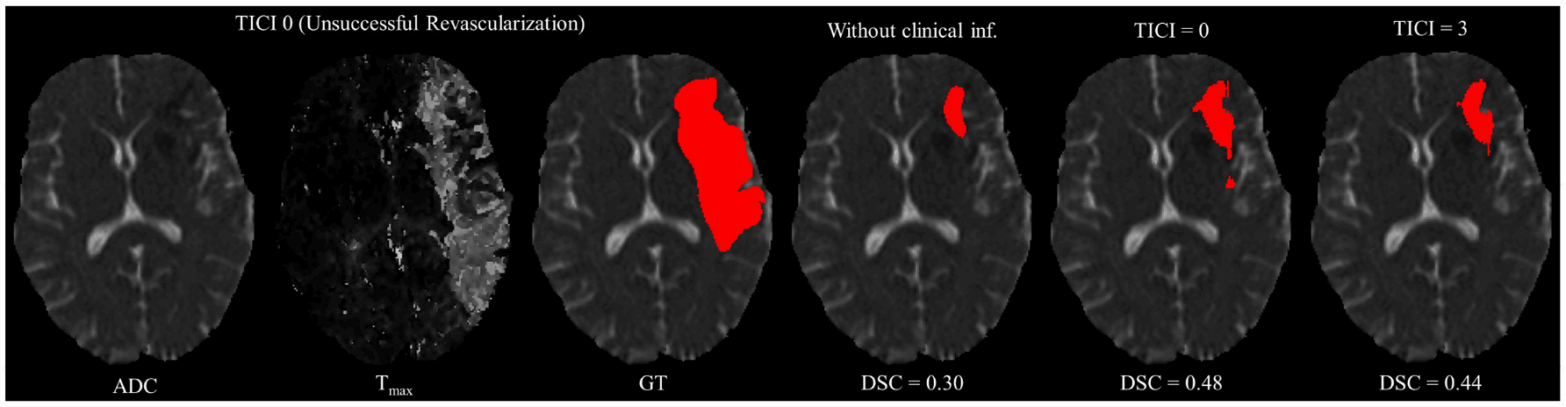
Example case of stroke lesion outcome prediction, with and without non-imaging clinical information in a patient with unsuccessful reperfusion. For sake of description we present the ADC and Tmax maps and the GT. In the presence of clinical information, we show the two possible outcomes: unsuccessful (TICI = 0) and successful reperfusion (TICI = 3), respectively.

**Figure 5:**
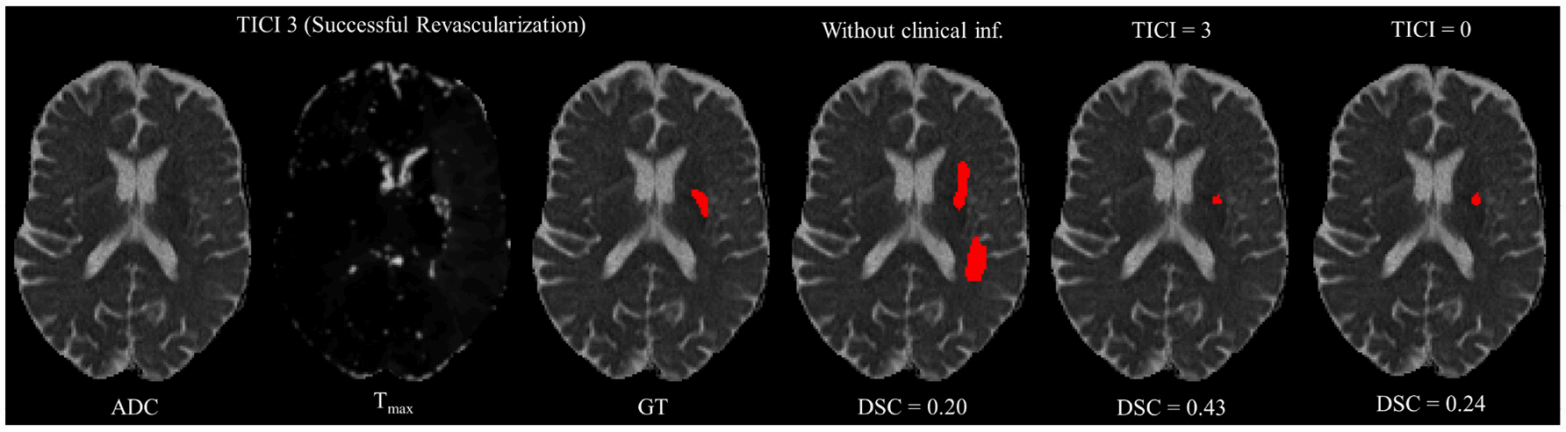
Example case of stroke lesion outcome prediction, with and without non-imaging clinical information in a patient with successful reperfusion. We also present the ADC and Tmax maps and the GT. In the presence of clinical information, we show the two possible outcomes: successful (TICI = 3) and unsuccessful reperfusion (TICI = 0), respectively.

However, in clinical practice the TICI score is only obtained after recanalization. Being so, predicting the stroke lesion at a 90 day follow up, during the sub-acute phase, needs to consider different reperfusion scenarios. In our proposal, we grant such property at patient-level domain. By adding an extra input channel that contains the TICI score, we aim to obtain tissue outcome predictions with successful and unsuccessful reperfusions. When accessing both case scenarios, during the decision-making process, our method could provide to clinicians additional information on the salvaged tissue if mechanical thrombectomy was performed. In Figures 4, 5 we show the added value of incorporating clinical information on two patients with different TICI scores: one with an unsuccessful reperfusion (TICI = 0), and one with a successful reperfusion (TICI = 3).

For each case, we present the tissue outcome predictions with and without non-imaging clinical information. In the absence of the TICI score, the tissue outcome prediction performs worse than our proposal, for both cases. Our proposal is capable of employing the TICI score to yield better predictions, which are corroborated by higher Dice scores, but also provides a result that physiologically is more plausible. Observing the stroke lesion outcome predictions of our proposal against the baseline, it is noticeable the presence of physiologically infeasible isolated regions in the latter. Additionally, we also tested if our method was capable of predicting different lesion outcomes by changing the TICI score. When changing the TICI score, we obtained different lesion outcomes for each patient. Furthermore, such scenarios agreed with the expected outcome describe for each TICI score (e.g., by changing from a TICI score of 3 to 0 it was observed a larger lesion outcome volume). From the latter study, we show that our proposal gained awareness to scenarios of no-perfusion and complete perfusion. Such capability could provide the clinicians useful insight on the benefits and risks associated to the mechanical thrombectomy. Moreover, it can also be used to forecast recovery, which is important for patient treatment and the complete standard care associated to patient recovery. To corroborate our qualitative analysis, Table 3 contains the ground-truth lesion volume for each case, alongside the predicted volume outcome for the original TICI score and for the opposite case scenario, respectively.

**Table 3:** Results obtained by our proposal on two patient cases with different TICI scores, alongside the obtained result after changing the original TICI score to its opposite (marked with a *).

**Case**	**GT volume (voxels)**	**TICI**	**Dice**	**Precision**	**Recall**	**Predicted volume (voxels)**
24	21,310	0	0.48	0.87	0.33	8170
		3*	0.44	0.90	0.29	6840
42	288	3	0.43	0.59	0.33	163
		0*	0.24	0.17	0.39	651

On Table 3, we show the effect of the TICI score in our proposal. When changing the TICI score we observe different stroke lesion outcome predictions, in agreement to the reperfusion success. When increasing the TICI score the volume of salvaged hypo-perfused tissue becomes higher, which corresponds to a stroke lesion shrinkage. Case 24, with *TICI* = 0, shows such behavior. After increasing the TICI score to *TICI* = 3, we obtain a smaller stroke lesion volume. As for case 42 with *TICI* = 3, when we decrease the TICI score from *TICI* = 3 to *TICI* = 0 the prediction volume characterized the opposite phenomena. With *TICI* = 0 there is higher hypo-perfused tissue loss, and the tissue outcome prediction volume is larger. From both case scenarios, the observed changes in the tissue outcome prediction volume shows that the TICI score was capable of driving the tissue outcome prediction scenario, and simultaneously grant a lesion growth or shrinkage in accordance with the physiological dynamics of each TICI score and without infeasible isolated regions.

### 4.2. ISLES 2017 Testing Set

In Table 4, we compare our proposal with methods from ISLES 2017 testing dataset, evaluated by the online platform (29) and ordered decreasingly by the DSC score. To reinforce our analysis, we also included the baseline method.

**Table 4:** Results of ISLES 2017 testing dataset, alongside our baseline method and proposal. Each metric contains the average ± standard deviation.

		**Dice**	**Hausdorff distance**	**ASSD**	**Precision**	**Recall**
Challenge	Mok et al. ^*^	0.32 ± 0.23	40.74 ± 27.23	8.97 ± 9.52	0.34 ± 0.27	0.39 ± 0.27
	Kwon et al. ^*^	0.31 ± 0.23	45.26 ± 21.04	7.91 ± 7.31	0.36 ± 0.27	0.45 ± 0.30
	Bertels et al. ^*^	0.30 ± 0.21	33.85 ± 16.82	6.81 ± 7.18	0.34 ± 0.26	0.51 ± 0.32
	Monteiro et al. ^*^	0.30 ± 0.22	46.60 ± 17.50	6.31 ± 4.05	0.34 ± 0.27	0.51 ± 0.30
	Lucas et al. ^*^	0.29 ± 0.21	33.85 ± 16.82	6.81 ± 7.18	0.34 ± 0.26	0.51 ± 0.32
	Choi et al. ^*^	0.28 ± 0.22	43.89 ± 20.70	8.88 ± 8.19	0.36 ± 0.31	0.41 ± 0.31
	Robben et al. ^*^	0.27 ± 0.22	37.84 ± 17.75	6.72 ± 4.10	0.44 ± 0.32	0.39 ± 0.31
	Pisov et al. ^*^	0.27 ± 0.20	49.24 ± 32.15	9.49 ± 10.56	0.31 ± 0.27	0.39 ± 029
	Niu et al. ^*^	0.26 ± 0.20	48.88 ± 11.20	6.26 ± 3.02	0.28 ± 0.25	0.56 ± 0.26
	Sedlar et al. ^*^	0.20 ± 0.19	58.30 ± 20.02	11.19 ± 9.10	0.23 ± 0.24	0.40 ± 0.29
	Rivera et al. ^*^	0.19 ± 0.16	63.58 ± 18.58	11.13 ± 7.89	0.27 ± 0.25	0.21 ± 0.17
	Islam et al. ^*^	0.19 ± 0.18	64.15 ± 28.51	14.17 ± 15.80	0.29 ± 0.28	0.25 ± 0.25
	Chengwei et al. ^*^	0.18 ± 0.17	65.95 ± 25.94	9.22 ± 6.99	0.37 ± 0.30	0.21 ± 0.23
	Yoon et al. ^*^	0.17 ± 0.16	45.23 ± 19.14	12.43 ± 11.01	0.23 ± 0.27	0.36 ± 0.32
	Baseline	0.24 ± 0.20	53.29 ± 26.95	10.59 ± 4.98	0.27 ± 0.27	0.50 ± 0.35
	Proposal	0.29 ± 0.22	47.17 ± 22.13	7.20 ± 4.14	0.26 ± 0.23	0.61 ± 0.28

* *Static results in Ischemic Stroke Lesion Segmentation Challenge (29)*.

Incorporating clinical information through the proposed custom loss function and the extra TICI channel resulted in a higher performance, in comparison to the baseline. Our proposal was able extract information from non-imaging data and to drive its training and testing phases toward better predictions. Therefore, the simultaneous incorporation of the reperfusion status, as an additional feature and in the loss function, improved performance of the classifier. In addition, we show the higher generalization capability of our proposal, since the performance metrics or our proposal for both datasets present less variation.

Although a previous work (15) had investigated the use of non-imaging clinical information to conduct the training of machine learning methods, such information has not been evaluated directly in the context of deep learning methods. The results on the ISLES2017 indicate the benefits of incorporating non-imaging clinical information in deep learning architectures implicitly during the training phase, and explicitly by extra channels, incorporating patient-specific information.

**Figure 6:**
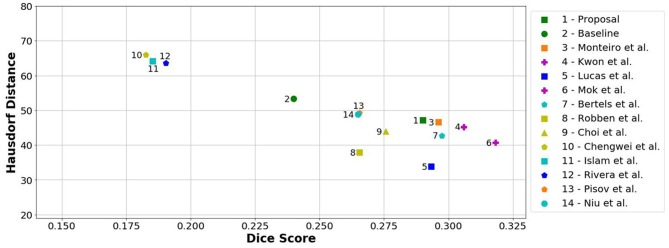
Hausdorff Distance vs. Dice score from methods of ISLES 2017 in the testing database. Note that closer to the horizontal axis and further away from the origin is better (i.e., high Dice and low Hausdorff). Ensemble methods are marked with a purple plus.

When comparing to the state-of-the-art methods, our proposal can reach competitive results, being placed among top scoring methods. With single model method, our proposal yields results within the top five methods, alongside ensemble approaches [e.g., Choi et al. (17)]. In the same group, our method achieved the highest recall metric, with lower precision score. As for the distance metrics, our proposal can provide competitive ASSD score, with low standard deviation, and a Hausdorff Distance among of top methods. We emphasize that, as post-processing step, our method only applies a simple morphological removal of small connected components. Therefore, elaborate schemes of post-processing such as Conditional Random Fields or even weighted schemes of ensemble can boost the performance of such approaches. Even in such cases, our approach provides a good robustness and precision in stroke lesion outcome delineation. To enforce such analysis in Figure 6, we show the average DSC score and the Hausdorff Distance obtained by each state-of-the-art method in ISLES 2017 testing dataset. Besides our proposal, we included the baseline method.

From Figure 6, we can observe the performance boost of our proposal over the baseline method, placing it within the group of top scoring methods.

## 5. Conclusions

Prediction of stroke lesion outcome has the potential to assist interventionists when assessing the risks and benefits associated to mechanical thrombectomy. Therefore, having such tool can provide useful information during the clinical decision process.

In this work, we propose a novel deep learning architecture that beyond previously proposed architectures incorporates clinical information in a principled way. To do so, our proposal integrates clinical information at two different levels of the architecture. The first level considers the population domain-knowledge, achieved through the development of a custom loss function, to depict relationships between the TICI score and the tissue outcome prediction. The second level considers the patient-specific domain, where the TICI is encoded into an input channel of the architecture. From the latter level, we showed that our proposal was able to characterize different outcome scenarios of successful and unsuccessful reperfusion. Such methodology presents itself as a ground-breaking tool with potential to access the risks and benefits associated to the mechanical thrombectomy. The evaluation of our proposal was conducted on the publicly available ISLES 2017 dataset. We observe that the proposed method has benefited from the combination of imaging and non-imaging information. In addition, when comparing to the state-of-the-art methods, we observed that a single architecture with fewer parameters, such as ours, yields competitive performance metrics similar to more elaborate and/or ensemble methods.

However, there is still room from improvement since none of the current state-of-the-art methods, provides the robustness and accuracy needed for clinical practice, and are currently bellow the inter-rater performance of expert radiologists (DSC=0.58) (19). In the future, we would like to investigate on adding other clinical information, such as TTT and TSS. We esteem that the proposed approach can be further applied to other diseases where clinical information complements imaging information.

## Ethics Statement

The study utilizes anonymized data from the Bernese stroke registry, a prospectively collected database approved by the Kantonale Ethikkomission Bern. All patients were treated for an acute ischemic stroke at the University Hospital of Berne between 2005 and 2013. The study was performed according to the ethical guidelines of the Canton of Bern (Swiss Humanforschungsgesetz) with approval of our institutional review board (Kantonale Ethikkomission Bern). Some cases were supplied by the University Medical Center Schleswig-Holstein in Lübeck, Germany. They were acquired in diagnostic routine with varying resolutions, views, and imaging artifact load. A smaller group of cases were scanned at the Department of Neuroradiology at the Klinikum rechts der Isar in Munich, Germany. Both centers are equipped with 3T Phillips systems. The local ethics committee approved their release under Az.14-256A. Full data anonymization was ensured by removing all patient information from the files and the facial bone structure from the images.

## Author Contributions

AP is the main author of the research presented in the manuscript, being supervised by RM during an internship at Bern. CS, VA, RW and RM gave thoughtful insights during this research.

### Conflict of Interest Statement

The authors declare that the research was conducted in the absence of any commercial or financial relationships that could be construed as a potential conflict of interest.
